# Colibactin Exerts Androgen-dependent and -independent Effects on Prostate Cancer

**DOI:** 10.1016/j.euo.2024.10.015

**Published:** 2024-11-14

**Authors:** Raag Agrawal, Sarah Al-Hiyari, Rupert Hugh-White, Robert Hromas, Yash Patel, Elizabeth A. Williamson, Mohammed F.E. Mootor, Alfredo Gonzalez, Jianmin Fu, Roni Haas, Madison Jordan, Brian L. Wickes, Ghouse Mohammed, Mao Tian, Molly J. Doris, Christian Jobin, Kevin M. Wernke, Yu Pan, Takafumi N. Yamaguchi, Seth B. Herzon, Paul C. Boutros, Michael A. Liss

**Affiliations:** a Department of Human Genetics, University of California-Los Angeles, Los Angeles, CA, USA; b Department of Urology, University of California-Los Angeles, Los Angeles, CA, USA; c Jonsson Comprehensive Cancer Center, University of California-Los Angeles, Los Angeles, CA, USA; d Institute for Precision Health, University of California-Los Angeles, Los Angeles, CA, USA; e Division of Hematology and Medical Oncology, Department of Medicine and the Mays Cancer Center, University of Texas Health-San Antonio, San Antonio, TX, USA; f Department of Microbiology and Immunology, University of Texas Health-San Antonio, San Antonio, TX, USA; g Department of Microbiology, Immunology, and Molecular Genetics, University of Texas, San Antonia, TC, USA; h Office of Health Informatics and Analytics, University of California-Los Angeles, Los Angeles, CA, USA; i Department of Urology, University of Texas Health-San Antonio, San Antonio, TX, USA; j Departments of Medicine, Infectious Diseases and Immunology, and Anatomy and Cell Physiology, University of Florida, Gainesville, FL, USA; k Department of Chemistry, Yale University, New Haven, CT, USA; l Department of Pharmacology, Yale School of Medicine, New Haven, CT, USA; m Department of Medical Biophysics, University of Toronto, Toronto, Canada

**Keywords:** Anaerobic bacteria, Cancer progression, Metagenomic sequencing, Metastases, Microbiota, Microbiome, Prognosis, Prostate cancer, Urine, 16S ribosomal amplicon sequencing

## Abstract

**Background and objective:**

The etiology of prostate cancer (PC) is multifactorial and poorly understood. It has been suggested that colibactin-producing *Escherichia coli* positive for the pathogenicity island *pks* (*pks*^+^) initiate cancers via induction of genomic instability. In PC, androgens promote oncogenic translocations. Our aim was to investigate the association of *pks*^+^ *E. coli* with PC diagnosis and molecular architecture, and its relationship with androgens.

**Methods:**

We quantified the association of *pks*^+^ *E. coli* with PC diagnosis in a volunteer-sampled 235-person cohort from two institutional practices (UT San Antonio). We then used colibactin 742 and DNA/RNA sequencing to evaluate the effects of colibactin 742, dihydrotestosterone (DHT), and their combination in vitro.

**Key findings and limitations:**

Colibactin exposure was positively associated with PC diagnosis (*p* = 0.04) in our clinical cohort, and significantly increased replication fork stalling and fusions in vitro (*p* < 0.01). Combined in vitro exposure to colibactin 742 and DHT induced more somatic mutations of all types than exposure to either alone. The combination also elicited kataegis, with a higher density of somatic point mutations. Laboratory analyses were conducted using a single cell line, which limited our ability to fully recapitulate the complexity of PC etiology.

**Conclusions and clinical implications:**

Our findings are consistent with synergistic induction of genome instability and kataegis by colibactin 742 and DHT in cell culture. Colibactin-producing *pks*^+^ *E. coli* may plausibly contribute to PC etiology.

**Patient summary:**

We investigated whether a bacterial toxin that is linked to colon cancer can also cause prostate cancer. Our results support this idea by showing a link between the toxin and prostate cancer diagnosis in a large patient population. We also found that this toxin causes genetic dysfunction in prostate cancer cells when combined with testosterone.

## 1. Introduction

Prostate cancer (PC) is a complex, heterogeneous disease and is the second most common cancer among men in the USA [1]. While there has been much research into how PC progresses and metastasizes, our understanding of its origins remains limited. The most substantial risk factors for PC are genetics, race, and age; other factors influencing its origins are poorly characterized [2,3].

Previous research has indicated that bacterial colonization may be a contributing cause of cancer [4–6]. For example, the bacterial metabolite colibactin has been linked to colorectal cancer [4,7,8]. Colibactin is a polyketide nonribosomal peptide produced by *Escherichia coli* and other Enter-obacteriaceae possessing a 54-kbp gene island referred to as polyketide synthase (*pks* or *clb*) [8,9]. In epithelial cell lines, *pks*^+^ *E. coli* induces DNA crosslinks and double-strand DNA breaks; in murine models, *pks*^+^ *E. coli* increases tumor invasion [10].

Preceding urinary tract infections, *pks*^+^ *E. coli* can translocate from the colon through the urethra and into the urinary tract [11,12]. Urinary tract infection, often with concomitant prostate inflammation, occurs more commonly after the age of 50 yr, just as PC incidence begins to rise. It has been hypothesized that repeat inflammation contributes to proliferative inflammatory atrophy [13], with the occurrence of *TMPRSS2:ERG* oncogenic gene fusions [14,15]. High spermidine content in the prostate induces the production of colibactin by *pks*^+^ *E. coli* [16]. Colibactin is thus a potential mechanistic link between bacterial infection and *TMPRSS2: ERG* fusion via induction of chromosomal breaks [14].

Colibactin mutational signatures are detected in normal tissue, suggesting that one or more additional insults are necessary for carcinogenesis [7]. Dihydrotestosterone (DHT) is an androgen involved in PC tumorigenesis and is associated with the pathophysiology of castration-resistant PC [17]. DHT induces nonhomologous chromosomal translocations via androgen receptor (AR) activation [18]. We hypothesized that colibactin and DHT have a synergistic effect via colibactin-induced double-stranded breaks, making AR-induced chromosome translocation more likely. The combination of colibactin and DHT might thus induce a higher rate of formation of oncogenic *TMPRSS2:ERG* fusions and potentially of other somatic variants.

To explore these potential mechanisms, we evaluated the association of *pks*^+^ *E. coli* with PC incidence in a 235-person cohort. We then used colibactin 742, a stable colibactin derivative [19,20], and DNA and RNA sequencing to evaluate the effects of colibactin, DHT, and their combination in vitro. The results demonstrate that combination treatment induces genomic instability and upregulates tumorigenic genes. These studies are consistent with the hypothesis that colibactin can synergize with DHT to promote PC tumorigenesis.

## 2. Methods

### 2.1. Study design and population

We conducted a retrospective analysis of prospective observational cohorts of individuals at risk of PC from whom a fecal sample was collected. We obtained fecal samples from two populations.

#### 2.1.1. SABOR cohort

The SABOR cohort is part of the San Antonio Biomarkers of Risk (SABOR) initiative, which is an 18-yr prospective study (institutional review board reference HSC20000030H). The cohort includes 4174 participants who are part of a regular regimen for serum prostate-specific antigen (PSA) testing, contributing to the clinical validation site for the National Cancer Institute Early Detection Research Network. The SABOR cohort represents a patient population typically at risk of PC and participating in community-based PC screening. These individuals did not routinely undergo biopsy unless recommended by their community physician, thus classifying them as at low risk of PC. Participants attend annual screenings at our dedicated SABOR clinics, where they consented to research procedures including collection of rectal swabs.

#### 2.1.2. PSA cohort

The second group, referred to as the PSA cohort (ie, prebiopsy), consisted of men included in a genitourinary repository (institutional review board reference HSC20050234H). These participants were scheduled to undergo prostate biopsy, which was the sole criterion for inclusion in the study; no other exclusion criteria were applied. Rectal swabs were collected during the biopsy scheduling process as part of an antibiotic selection protocol for infection prevention. Notably, patients were not on antibiotic therapy at the time of swab collection, which typically occurs on the morning of the biopsy. The standard collection process involves two swabs; hence, the second, unused swab was preserved, anonymized, and designated for bacterial DNA isolation within our genitourinary biospecimen bank. Consent was deemed unnecessary for this cohort since the specimens were gathered for repository purposes, posed no risk to the patients, and were deidentified, and data collection was managed by an independent third party.

### 2.2. Outcomes

Our primary outcome was prediction of International Society of Urological Pathology grade group ≥2 (Gleason ≥ 3 + 4) PC. Our secondary outcome was any PC, defined as grade group ≥1. The primary predictor of PC is the microbiome score.

### 2.3. Sample collection and preparation

All specimens were collected prospectively, especially before antibiotic use in the prebiopsy PSA cohort. We used both rectal swabs and glove tips for fecal microbiome collection. In previous investigations, we found that both methods are highly correlated with results and DNA yield [21]. For collection of a rectal swab, the urology provider used sterile Medline E-Z Lubricating Jelly and placed the swab approximately 2 inches into the rectum and turned it 360°. The swab was then removed and examined for stool. The physician reinserted the swab if no material had been collected. The swab was then placed in a 15-ml sterile centrifuge tube containing 1 ml of phosphate-buffered saline (PBS) and stored at 4 °C during transport to the laboratory within 4 h of collection. Swab and PBS material was then stored at −20 °C until DNA isolation.

### 2.4. DNA isolation and sequencing

For sample input, we attempted to obtain a visible amount of stool on the glove tips and swabs. DNA was isolated from fecal samples using our standard operating procedure [22]. Genomic DNA was purified from fecal samples using a QIAamp Fast DNA Stool Mini Kit according to the kit protocol (Qiagen, Germantown, MD, USA). The DNA concentration was measured using a NanoDrop instrument (Thermo Scientific, Waltham, MA, USA). Polymerase chain reaction (PCR) for sequencing used primers for *clbN* (Supplementary Table 3). The amplicon size was 733 bp for colibactin N (*clbN*) and 579 bp for colibactin B (*clbB*). Primers were selected from previous studies [23,24]. A subject was classified as *pks*^+^ and capable of producing colibactin if the result for either *clbN* or *clbB* was positive.

### 2.5. Cell culture and colibactin treatment

RWPE-1 cells, a nontumorigenic line derived from normal human prostate epithelium, were used as an in vitro benign model for the prostate [25]. RWPE-1 cells retain many of the characteristics of normal epithelial cells and have intact AR activation, matching almost all primary PCs [25,26]. Cells were obtained from ATCC (Manassas, VA, USA; catalog no. CRL-3607) and were cultured using a keratinocyte serum-free media kit (Life Technologies, Carlsbad, CA, USA; catalog no. 17005–042), which includes bovine pituitary extract (BPE) and EGF. The final concentrations in the medium were 0.05 μg/ml BPE and 5 ng/ml EGF. Parallel cultures were grown in the presence or absence of 10 nM dihydroxytestosterone (DHT; Millipore Sigma, Burlington, MA, USA; catalog no. A8380). An important caveat to our molecular studies is our use of colibactin 742 as a synthetic substitute for colibactin. Owing to several facile modes of degradation, isolation and study of colibactin have been impractical thus far. Wernke et al [19] found that the C36–C37 1,2-diketone bond is critical to the instability of colibactin and developed a synthetic molecule that retains the ability to form DNA interstrand crosslinks, analogous to those produced by *clb*^+^ bacteria. Exposure of *Galleria mellonella* larvae to colibactin 742 induced greater intestinal DNA damage than an inactive variant, as assessed by comet assay, demonstrating in vivo genotoxicity [20]. Furthermore, colibactin 742 reproduces many features of the bacterial phenotype, including induction of the transcription of DNA damage response genes such as *p53* in colonic epithelial cell lines [20].

For the experiments described here, RWPE-1 cells were seeded at 10^5^ cells per 10-cm dish. Cells were treated with plasmocin for 2 wk when first cultured. Three conditions were tested. In the absence of DHT, cells were incubated with either (1) 1 μM colibactin 742 for 24 h or (2) 10 μM colibactin 742 for 7 d, after which the colibactin-containing medium was replaced with fresh medium and the cells were allowed to grow and recover for a further 14 or 7 d respectively. (3) In the presence of DHT, cells were treated with 10 μM colibactin for 7 d, followed by a 7-d recovery period. At the end of the recovery time, the cells were harvested and washed with PBS and the cell pellet was snap-frozen at −80°C before processing for down-stream analyses.

### 2.6. DNA fiber assays

DNA fiber analysis was carried out as previously described [27]. In brief, RWPE-1 cells were grown in six-well plates (2 × 10^5^ cells/well) using Keratinocyte Serum-free Medium (Invitrogen, Carslbad, CA, USA; Gibco catalog no. 17005–04) with or without 10 nM DHT. Cells proliferated slightly faster in DHT, otherwise there was little difference in cell behavior with or without DHT. Cells were pulse-labeled with 100 μM 5-iodo-2’-deoxyuridine (IdU) and incubated at 37°C for 20 min. After washing with fresh medium, colibactin was dissolved in dimethylsulfoxide (DMSO) and 10, 20 or 40 μM was added to the medium for 1 h. The colibactin-containing medium was removed, cells were washed once in medium, fresh medium containing 300 μM 5-chloro-2’-deoxyuridine (CldU) was added, and cells were further incubated for 20 min at 37 °C. Cells were harvested, washed with PBS, and resuspended in PBS at 2 × 10^5^ cells/ml. DNA fibers were processed using a FiberComb molecular combing system (GenomicVision, Bagneux, France). Cells were mixed with low-melting agarose and agarose plugs were prepared and chilled at 4 °C for 30 min to solidify the agarose. Each plug was mixed with 200 μl of 0.5 M EDTA, 25 μl of sarkosyl, and 50 μl of proteinase K and incubated at 50 °C for 18 h. Plugs were washed three times with TE buffer (10 mM Tris, pH 8, 1 mM EDTA) at room temperature and placed in a reservoir containing 1 ml of 2-(*N*-morpholino)ethanesulfonic acid (MES) buffer (pH 5.5). The reservoirs containing the plugs were incubated at 65 °C for 30 min to melt the agarose. The melted agarose was digested with 2 μl of agarase (New England Biolabs, Ips-wich, MA, USA) at 42 °C for 14–18 h. After digestion with agarase, the reservoirs were stored at 4 °C for 2–3 d before processing for DNA fibers on slides (GenomicVision) using the FiberComb molecular combing system. The newly synthesized CldU and IdU tracks were labeled (for 2.5 h in the dark at room temperature) with antibodies recognizing CldU and IdU, followed by 1- h incubation with secondary antibodies at room temperature in the dark. Slides were mounted in PermaFluor aqueous self-sealing mounting medium (Thermo Scientific), and images of DNA fibers were captured with a confocal Olympus FV1000D scanning microscope (Olympus America Inc., Center Valley, PA, USA). DNA fiber images were analyzed using ImageJ software. At least 200 tracks were quantified for each experiment. All experiments were repeated at least three times and data are expressed as the mean ± standard error (Supplementary Table 4).

### 2.7. Replication fork fusion and fork degradation assays

A modified DNA fiber analysis was performed to assess the fate of unrepaired replication forks after oxidative stress [27], including fusion with another unrepaired fork or degradation. When both IdU and CldU are present at the same time, the amount incorporated for one versus the other is governed by the frequency of the cognate opposite base for pairing. Thus, the green/red pattern within the same fiber when both labels are present at the same time is unique for each replication fork. This characteristic can be used to code individual replication forks. In addition, new replication forks that move away from the same origin exhibit approximately the same length of labeled fibers because they have been progressing for the same length of time. Inappropriate fusion of two collapsed replication forks would therefore result in asymmetrically labeled and asymmetrically sized forks adjacent to each other on a single DNA fiber. It is recognized that such unrepaired replication fork fusion (RFF) events will be a subset of such asymmetric fibers, as they can also reflect rescue of a stalled fork by an adjacent fork.

To measure unrepaired RFF events, RWPE-1 cells were grown in six-well dishes (1.5 × 10^5^ cells/well) as above. At 48 h after transfection, cells were pulse-labeled with 25 μM CldU for 30 min at 37 °C, and then 25 lM IdU was added without removing the CldU. Cells were incubated at 37 °C for 30 min, washed with fresh medium, and incubated in medium containing colibactin at 20 μM for 1 h. Cells were washed with fresh medium and allowed to grow for 2 h. The cells were then trypsinized, collected after centrifugation, and processed for DNA fiber analysis as described above, except CIdU was stained red and IdU green and fibers were stained with 4 ,6-diamidino-2-phenylindole to verify that adjacent tracks were on the same fiber. Images of the fraction of two adjacent asymmetrically labeled and asymmetrically sized forks on the same fiber in relation to the total number of forks were captured with a Nikon Eclipse Ti microscope (Nikon, Melville, NY, USA) and analyzed using ImageJ software. Representative images from >270 captured images in each condition from four independent experiments are shown. Data are the mean ± SE from four independent experiments (Supplementary Table 5).

### 2.8. DNA alignment and quality control

DNA libraries were prepared for whole-genome sequencing using a KAPA Hyper Prep Library Preparation kit and samples were sequenced at the UCLA Technology Center for Genomics and Bioinformatics. We conducted quality control on all FASTQ results using fastqc v0.11.8 and multiqc v1.13 [28,29]. Subsequent adapter trimming was performed using fastp v0.20.1 before alignment [30]. DNA alignment was performed using BWA-MEM2 v2.2.1 to GRCh38-BI-20160721 with alt-aware alignment [31]. We marked duplicates using GATK Picard MarkDuplicates [32]. The mean coverage depth across the genome was assessed using mosdepth v0.3.2, ignoring duplicates (Supplementary Fig. 1A–D) [33–35].

### 2.9. Calling of variants, functional annotations, and copy numbers

GATK v3.7.0 was used to perform indel realignment. Base quality-score recalibration for all samples was conducted using GATK v.4.2.3 [32]. Calling of germline single-nucleotide polymorphisms (SNPs) was performed for each sample alone using GATK HaplotypeCaller, applying variant quality-score recalibration to called SNPs and filtering ambiguous variants. We next called somatic single-nucleotide variants (SNVs) between tumor-normal pairs using Mutect2 v4.2.4.1 with the setting scatter_count = 50 [36]. Samples treated with DMSO only were used as a paired normal for all treatment conditions. Mutect2 identifies short somatic mutations such as SNVs and indels via local assembly of haplotypes. Annotation of somatic SNVs was performed using Annovar v20211016 [37]. Functional annotation was used to define SNVs as missense (nonsynonymous), nonsense (stop codon gained or lost), or splicing (splice donor or splice acceptor) variants. Somatic structural variants were called for each sample with a paired DMSO-only sample using Delly v1.1.5 with the settings map_qual = 20, min_clique_size = 5, and mad_cutoff = 15 [38]. Circos plots were produced for every pairwise combination using the *circos* v0.69.9 package for R [39]. We identified copy-number alterations (CNAs) using *batten-berg* v2.2.9 [40]. Subclones were identified as all regions called by *battenberg* with a *p* value <0.05. Total copy number was defined for both the trunk and subclones as the sum of the major-allele and minor-allele copy numbers from *battenberg* solution A. A total copy number of >2 was classified as a gain, a total copy number of <2 as a loss, and 2 as neutral. Genes were collated from the Ensembl annotation database and bedR v1.0.7 was used to determine whether genes overlapped regions with copy-number gains or losses [41].

### 2.10. RNA alignment and quantification

Transcriptome sequencing was performed using 100-bp paired-end sequencing and the TruSeq Stranded with Ribo-Zero Gold Library. We aligned RNA FASTQ files to GRCh38.13 using STAR v2.7.6a [42]. fastqc v0.11.8 generated reports for each sample for per-base sequence quality, per-base sequence content, GC content, and other metrics[28]. We used fastp v0.20.1 to trim adaptor sequences [30] and multiqc v1.13 to summarize fastqc metrics for all samples and collate the results into summary plots [29]. RNA quantification was performed using kallisto v0.46.0 [43]. The mean coverage depth was assessed using mosdepth v0.3.2, ignoring duplicates [33].

### 2.11. Analysis of differential mRNA abundance

We used edgeR v3.36.0 to analyze differential mRNA abundance [44]. Genes were filtered using a cutoff of 1 transcript per million (TPM). Trimmed mean of M-values (TMM) normalization was used to account for differences in library size between samples. Counts per million reads were generated on the basis of TMM-normalized values.

Two-way analysis of variance was run in edgeR for three conditions: colibactin, DHT, and the interaction colibactin: DHT for the combined treatment. The following equation describes the model used:

y=DHT+colibactin+colibactin:DHT.

We fitted a negative binomial generalized linear model (GLM) and conducted quasi-likelihood F tests to obtain *q* values for each gene in each comparison, with statistical significance set at *q* < 0.05. Statistically significant genes were analyzed for Gene Ontology (GO) enrichment using gprofiler2 v0.2.1 [45]. All genes with a result >1 TPM were compiled into a custom background list. All GO subontologies were included in gprofiler2 for analysis [46].

### 2.12. Gene fusions and splice isoforms

Gene fusions were identified using arriba v2.3.0 [47]; splice isoforms were identified and quantified using rMATS v4.1.2 (Supplementary Table 6) [48].

### 2.13. Statistical analysis

All statistical analyses were performed using R v4.2.2 [49]. The frequency and proportion were calculated for categorical data. The mean and standard deviation are reported for continuous variables with a normal distribution. Fisher’s exact test was used for comparison of results for categorical variables.

Biochemical recurrence (BCR) of PC was used as the primary endpoint for survival analysis. Genes and age were used as covariates in the Cox proportional-hazards models in the *survival* v3.3.1 package for R [50]. The hazard ratio (HR) and 95% confidence interval are reported. Statistical significance was set at a two-sided p value of ≤0.05. Data visualization was performed using the BPG v7.0.5 package [51].

### 2.14. Data availability

DNA and RNA sequencing data are available at the Sequence Read Archive under BioProject PRJNA990477.

## 3. Results

### 3.1. Colibactin exposure is associated with PC diagnosis

While *pks*^+^ *E. coli* that produce colibactin are associated with colorectal tumors, there have been few investigations into their potential role in cancers in other microbiota niches [8,52]. To assess the link between *pks*^+^ *E. coli* and PC tumorigenesis, we conducted a retrospective analysis of two observational cohorts of individuals at elevated risk of PC. The two clinical cohorts (PSA cohort and SABOR cohort) comprised a total of 620 individuals who we screened for *pks*^+^ *E. coli*. The prevalence of *pks*^+^ *E. coli* was 43.7% (160/366 patients) in the SABOR cohort and 41.7% (106/254 patients) in the longitudinal PSA cohort.

The clinical PSA cohort was used to test the association between *pks*^+^ *E. coli* and PC diagnosis. Table 1 lists demographic and clinical data for the subgroups with and without PC. An inverse relationship between diabetes and PC diagnosis has previously been reported [53]. There was no significant difference in diabetes prevalence between the subgroups. Colibactin exposure was defined as the presence of a *pks* gene island according to PCR. In our clinical cohort, colibactin exposure was significantly positively associated with PC diagnosis (odds ratio 1.74; *p* = 0.036, one-sided Fisher’s exact test). After removal of age and PSA outliers (Supplementary Table 7 and Supplementary Fig. 6A–D) this association remained significant. On stratification of patients according to US Preventative Service Task Force age groups (<55, 56–69, >70 yr), the association between *pks*^+^ *E. coli* and PC diagnosis remained consistent in magnitude across the subgroups (Supplementary Fig. 6F). There was no significant positive association between either cytolethal distending toxin or cytotoxic necrotizing factor 1 and PC diagnosis of any grade (Fig. 6C).

To determine if genotoxin exposure was associated with PC grade at diagnosis, we tested for differences in genotoxin presence (assessed via PCR) between grade group 1 and grade group ≥2 PC. There was no significant association for any genotoxin. We also investigated whether genotoxin exposure was more common for some grade groups than others and found no significant association (Fig. 6D).

Using National Comprehensive Cancer Network categories for PSA (<4, 4–10, >10 ng/ml), we investigated whether the association between *pks*^+^ *E. coli* and PC diagnosis was enriched in any PSA group. We found a very significant association (*p* = 0.0079) in the group with PSA 4–10 ng/ml (Supplementary Fig. 6E). There was no association in the other groups. Taken together, these results suggest that grade group is not dependent on genotoxin exposure, and genotoxin exposure is not enriched in PC tumors of higher grade group or with high PSA, pointing to a potential role of other risk factors in initial tumorigenesis.

### 3.2. Combination treatment induces genome-wide rearrangements

**Fig. 1 –:**
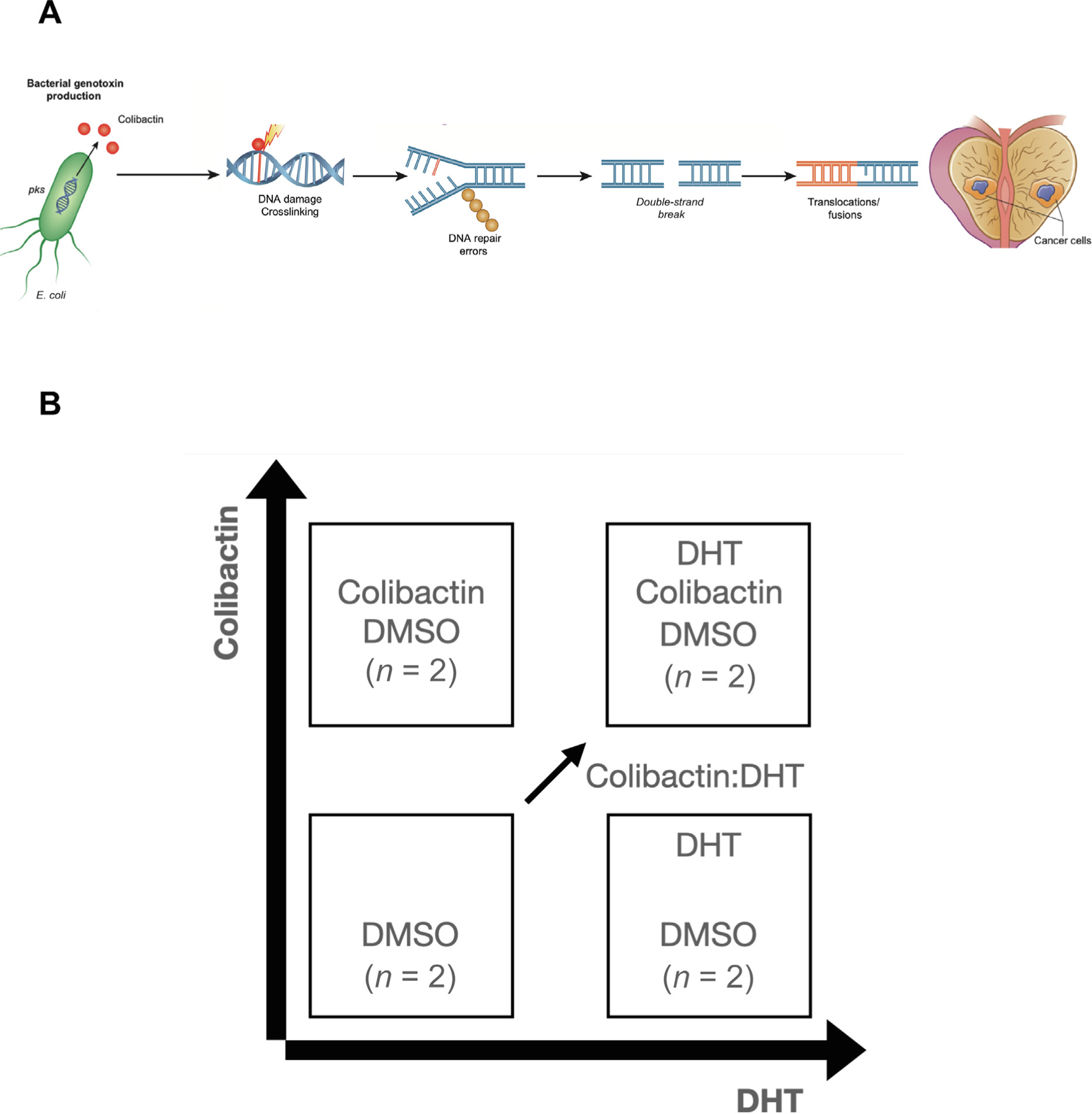
(A) Diagram of the mechanism by which colibactin may contribute to carcinogenesis in the prostate. (B) Experimental outline. DHT = dihydrotestos-terone; DMSO = dimethylsulfoxide.

**Fig. 2 –:**
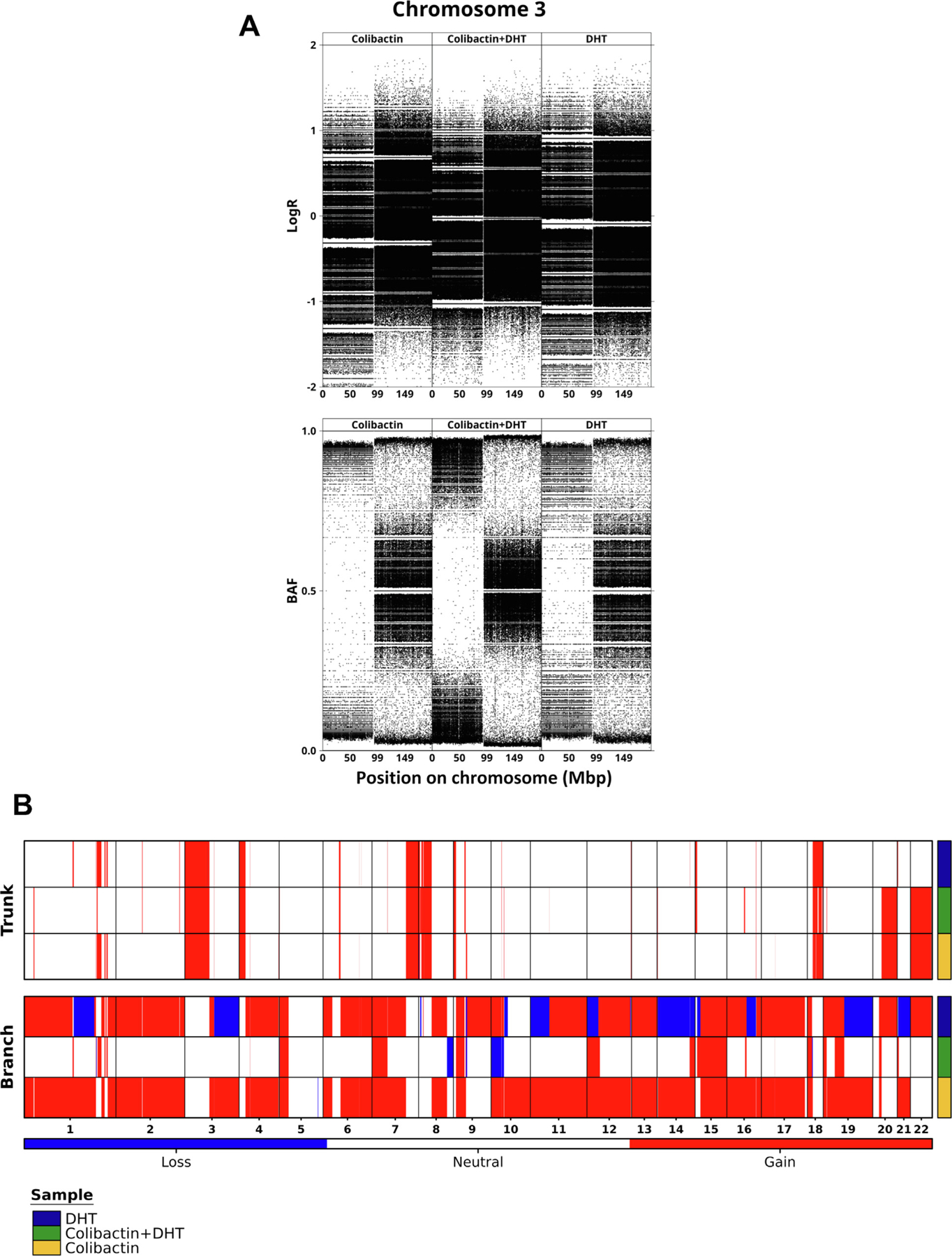
Copy number results. (A) Plots of logR and B allele frequency (BAF) by genome position. (B) Copy number landscape for all conditions by clonal population (branch, trunk). DHT = dihydrotestosterone.

We hypothesized that synergy between AR activation and colibactin genotoxicity (Fig. 1A) induces a variety of oncogenic pathways [54]. We exposed RWPE-1 cells to DHT, colibactin 742, and their combination (Fig. 1B). An increase in CNA burden is associated with shorter time to BCR and death [55,56]. Relative to DMSO, all three treatment conditions led to large segmental gains within chromosomes (Fig. 2A). Among the trunk cell populations, there were more genome-wide gains after colibactin 742 treatment than after DHT treatment alone (Fig. 2B). Between trunk populations, the combination treatment had large overlaps of CNAs with DHT-alone, while notably sharing several CNAs unique to colibactin 742-alone. Among the branch populations, similar high rates of copy number-gains were observed after colibactin 742 treatment and DHT treatment alone. This pattern of widespread gains throughout the genome suggests that both colibactin 742 and DHT are independently sufficient to induce whole-genome duplication events.

**Fig. 3 –:**
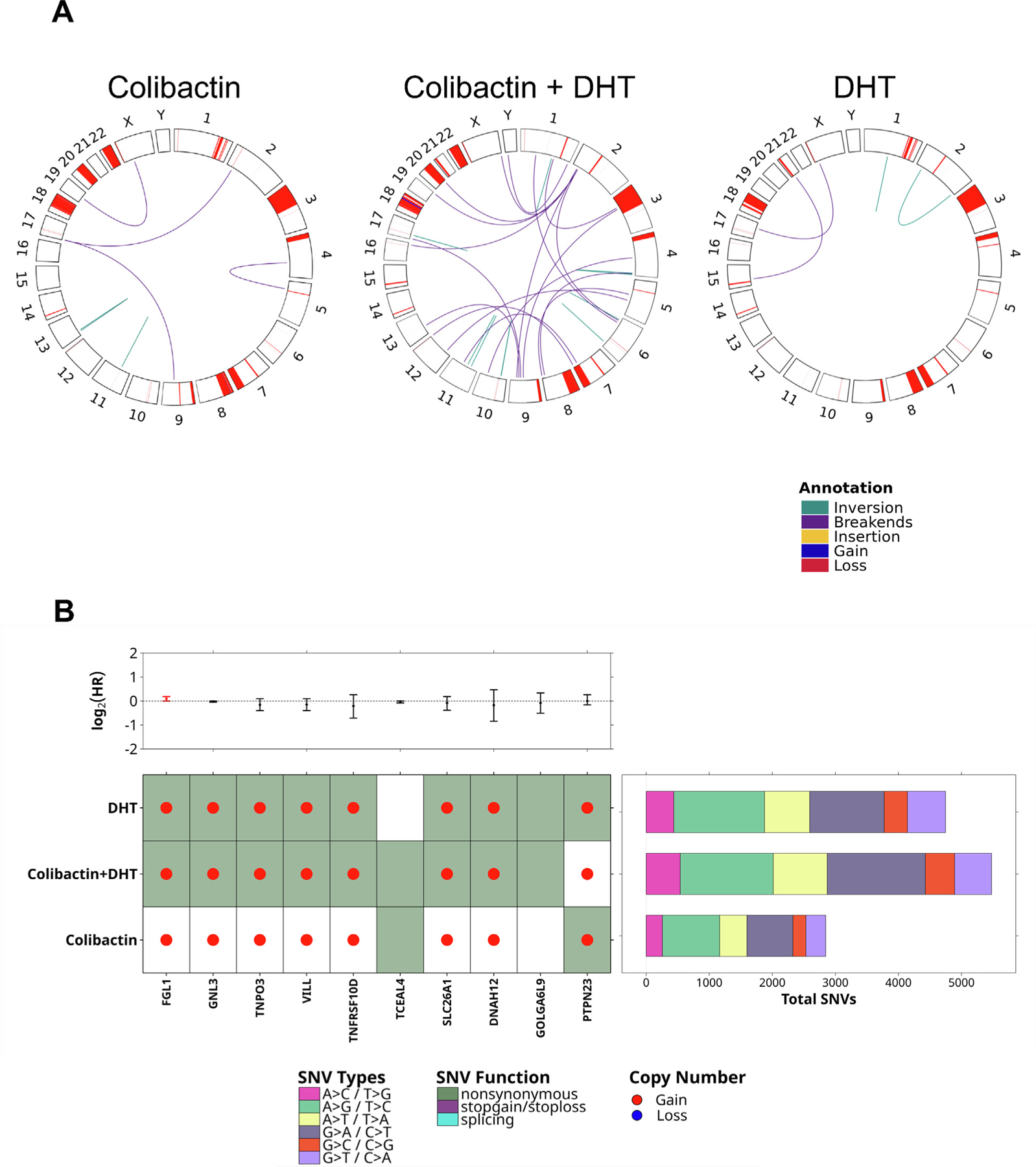
Genome instability in terms of SNVs and structural variants. (A) Comparison of structural variants by condition. (B) Summary of functionally annotated somatic SNVs and copy-number aberrations in all conditions, alongside gene-level HRs for biochemical recurrence (*p* < 0.05) and total SNV counts labeled by mutation type. SNV = single-nucleotide variant; HR = hazard ratio; DHT = dihydrotestosterone.

PC evolves widespread structural variation (SV), including inversions and insertions [57]. These features were quantified and reported relative to DMSO-only treatment. The combination treatment group had the largest number of SVs (Fig. 3A), while DHT and colibactin 742 individually yielded smaller and comparable increases in the number of SVs.

Somatic single-nucleotide variants (SNVs) can also be drivers of localized PC [58]. Combination treatment induced more SNVs than the other treatments (Fig. 3B). To quantify mutational density, the mean number of SNVs per million well-covered base pairs (>30×) was measured. Combination treatment induced 12.9 SNVs/Mbp, in comparison to 7.9 SNVs/Mbp with DHT and 3.8 SNVs/Mbp with colibactin 742 alone. Consistent with these findings, combination treatment induced 64 nonsynonymous SNVs, in comparison to 37 with DHT and 15 with colibactin 742 alone. Of the genes with somatic SNVs induced by the combination treatment, one (*FGL1*) is associated with BCR in the International Cancer Genome Consortium PRAD-CA data set (*n* = 142; Fig. 3B) [59].

### 3.3. Kataegis is present at higher rates after combination treatment

**Fig. 4 –:**
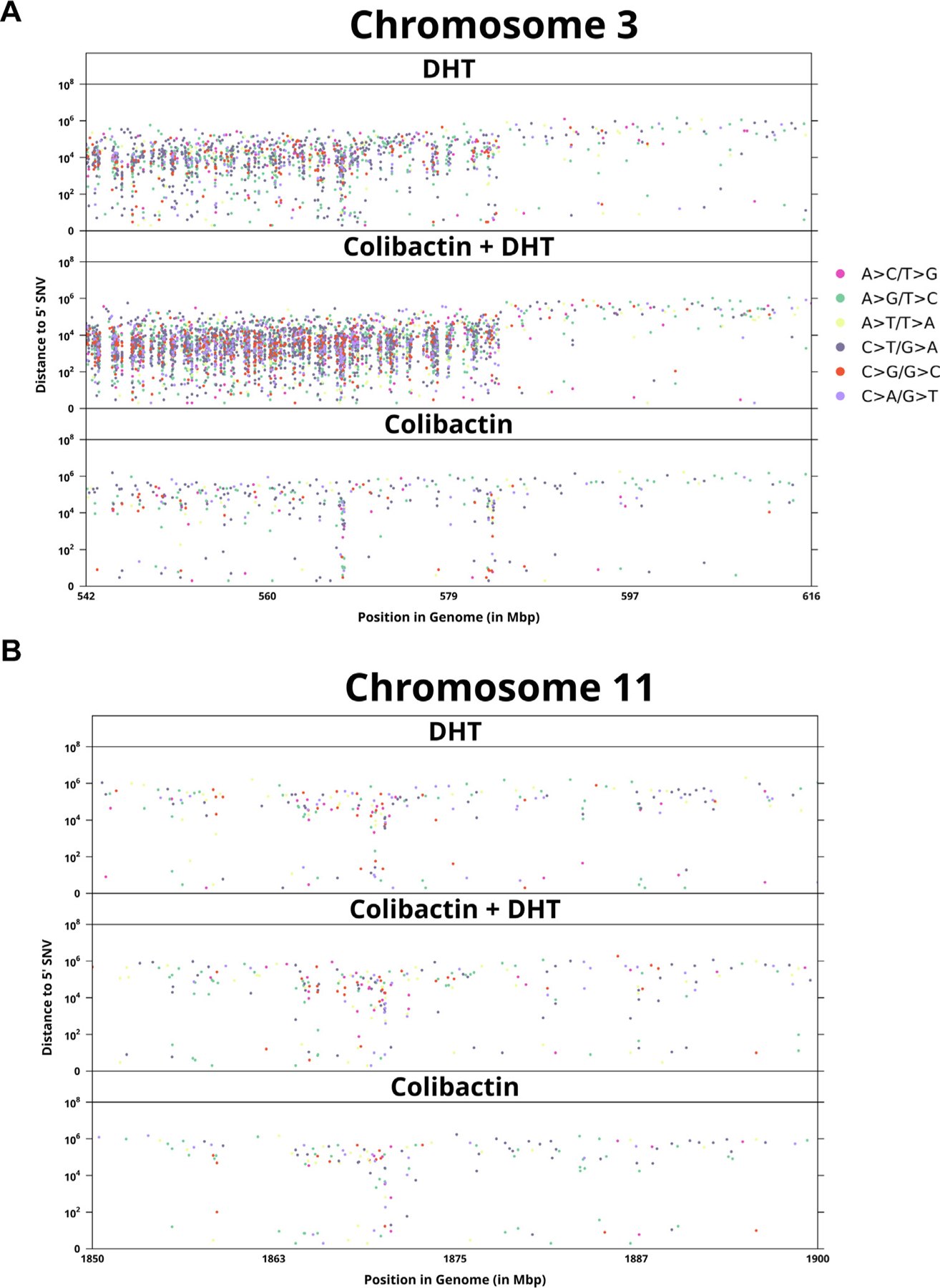
Kataegis events in (A) a chromosome with differential mutation rates by condition and (B) a chromosome for which there are no differences in kataegis events by condition. DHT = dihydrotestosterone; SNV = single-nucleotide variant.

Localized SNV hypermutation (kataegis) is present in ~25% of PC primary tumors [60]. Kataegis is associated with genomic instability, altered DNA repair, and deletions in chromatin-remodeling proteins [60]. We observed striking differences in the distribution and frequency of kataegis events at well-covered sites (>30×) across the three conditions. Combination treatment was associated with the most regions of kataegis and the greatest mutational density in shared hypermutated regions (Fig. 4A). This effect was only observed for hypermutated regions and not present at all sites (Fig. 4B). To quantify hypermutation, the genome was divided into 1-Mbp bins and the mean inter-SNV distance was calculated by bin. The median bin inter-SNV distance of 16 255 bp for combination treatment was substantially lower than the 72 399 bp for colibactin 742 and 23 611 bp for DHT. Similarly, when considering only variants from COSMIC, the median bin mean distance was lower for the combination treatment (135 311 bp) than for DHT (151 484 bp) and colibactin 742 (153 125 bp). This highlights the greater SNV density of somatic functional mutations genome-wide after combination treatment.

### 3.4. Combination treatment activates tumorigenic genes

**Fig. 5 –:**
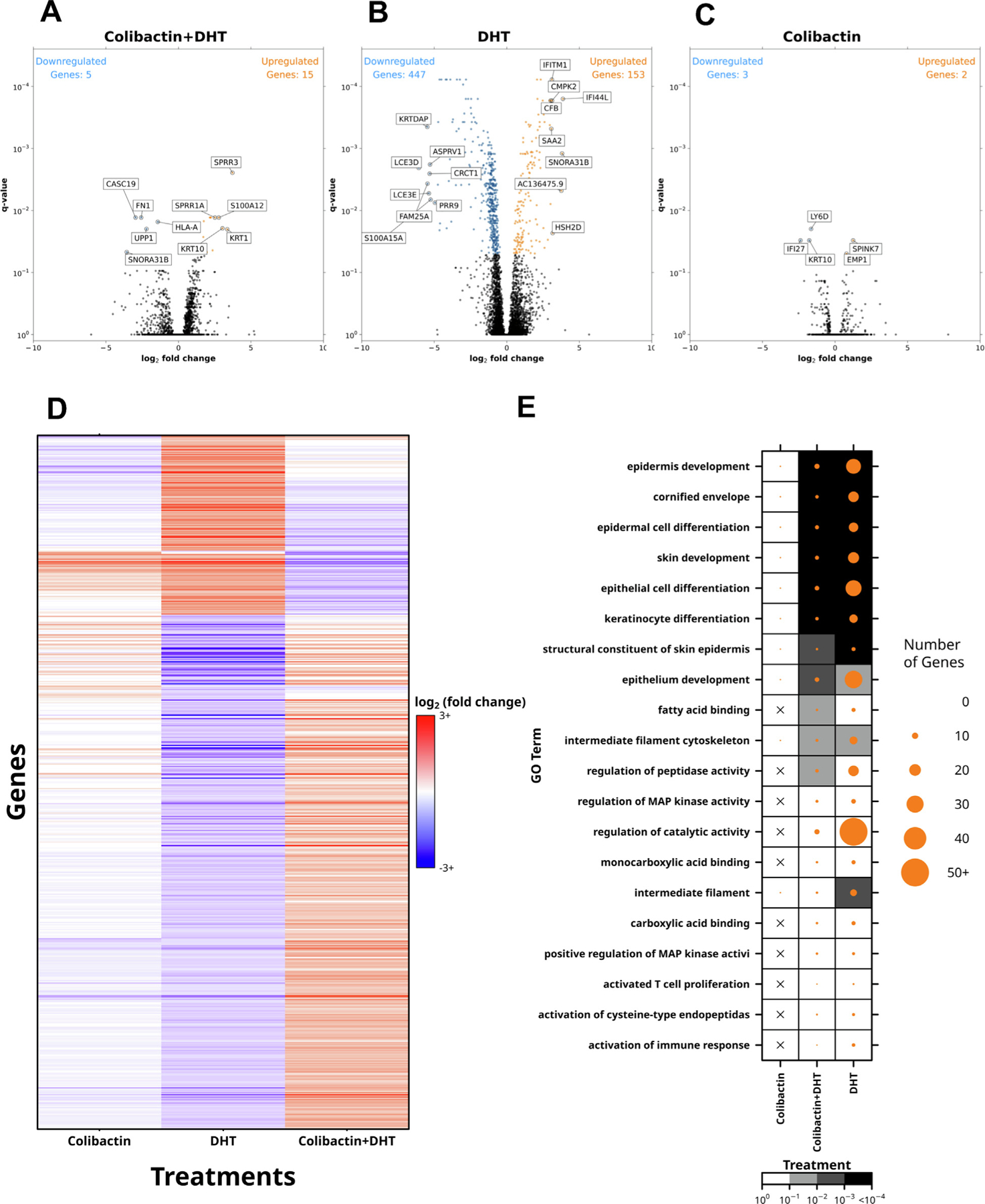
(A–C) Differential mRNA abundance; genes with significant (FDR < 0.05) and large (>1 absolute log_2_ fold change) are highlighted. (D) All genes identified as significant (FDR < 0.05) in any condition, clustered by log_2_ fold change. (E) GO terms in descending order by increasing *q* value and gene-set size by condition. DHT = dihydrotestosterone; FDR = false discovery rate; GO = Gene Ontology.

We analyzed RNA sequencing data to obtain an insight into the influence of colibactin 742 and DHT on gene abundance. We assessed differential mRNA abundance, alternative splicing (Supplementary Fig. 2A,B), and gene fusions (Supplementary Fig. 3A,B). To distinguish the effects of DHT, colibactin 742, and any potential synergy between them, we fitted a two-factor, two-level linear model to each transcript. As expected, exposure to DHT altered the abundance of many transcripts (447 downregulated; 153 upregulated; FDR < 0.05). By contrast, exposure to colibactin 742 altered the abundance of a very small number of transcripts (3 downregulated; 2 upregulated). Intriguingly, exposure to combined DHT and colibactin 742 altered the abundance of 20 transcripts (15 upregulated; 5 down-regulated; Fig. 5A–C and Supplementary Table 1). There was little overlap between these three groups of transcripts (Supplementary Fig. 4A,B). Several genes were upregulated by DHT alone, and this upregulation was suppressed by addition of colibactin 742 (Supplementary Table 2 and Supplementary Fig. 4C). To investigate any underlying structure affecting the differential mRNA abundance between conditions, we clustered genes that were significant for any of the three conditions. There were few overlaps in genes with significantly differential abundance as indicated by the log_2_ fold change (Fig. 5D).

GO analysis was performed to gain insight into the biological processes affected by the combination treatment. Significantly differentially abundant genes (FDR < 0.05) after combination treatment were significantly enriched in several GO terms related to epidermis development (FDR = 9 × 10^−9^), epidermal cell differentiation (FDR = 6.7 × 10^−6^), and keratinocyte differentiation (FDR = 1.4 × 10^−4^). We compared GO term enrichment for differentially abundant genes between conditions and observed considerable overlap (9/11 terms) of significant GO terms between the DHT-alone and combination conditions (Fig. 5E). GO terms for fatty acid binding and peptidase activity regulation were enriched only for transcripts showing DHT-colibactin 742 interactions.

### 3.5. Colibactin 742 induces stalling of replication forks

**Fig. 6 –:**
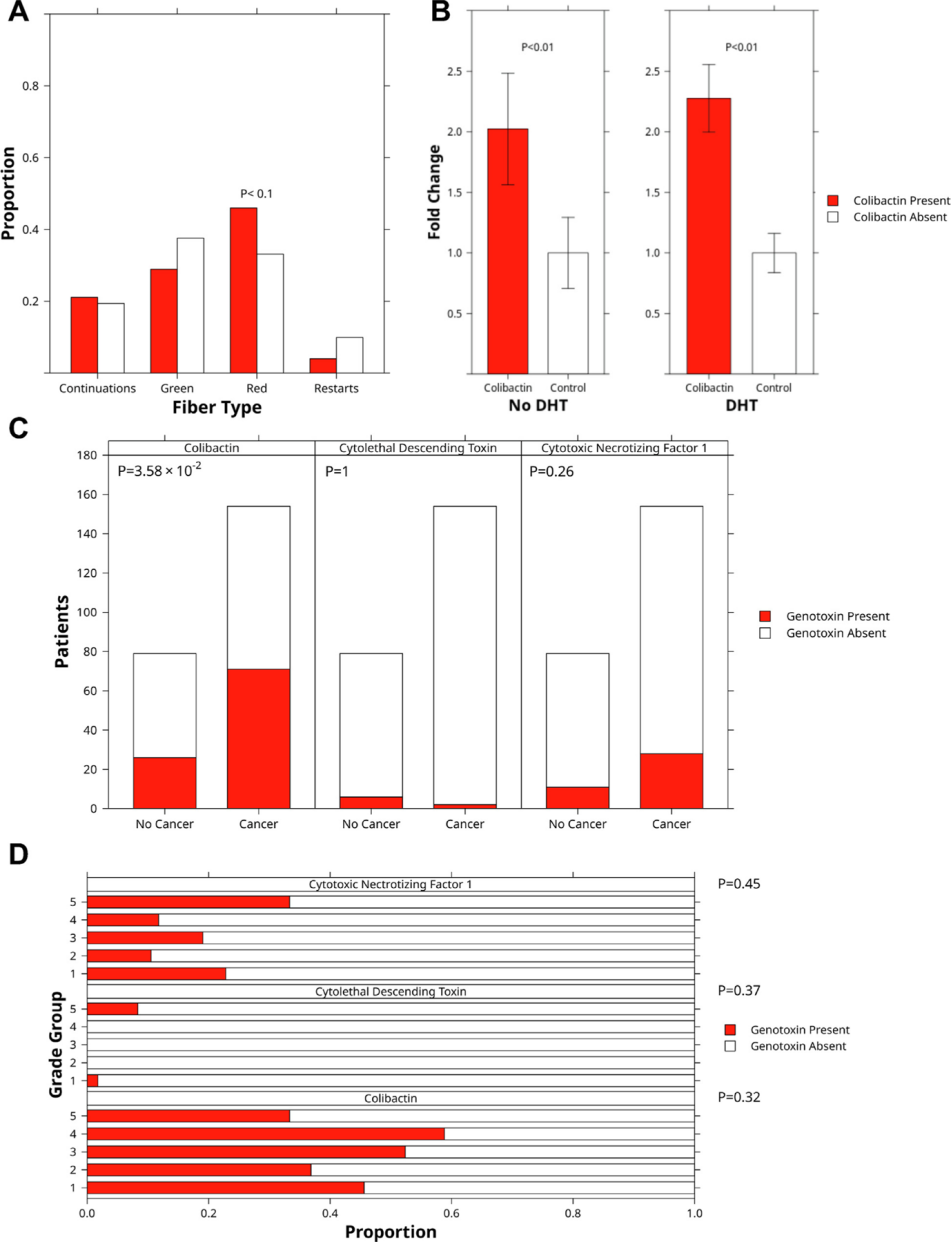
Clinical associations. (A) Proportion of fork types after treatment with DHT or colibactin at the 40-min time point. Red denotes stalled forks; green denotes restarted forks. Restarts are forks that only restarted after treatment. Continuations are unaffected forks. (B) Fold-change in fork fusions by treatment condition. (C) Association of genotoxin presence with cancer presence and grade in the prostate-specific antigen cohort (*n* = 235). (D) Proportion of patients testing positive for each genotoxin by International Society of Urological Pathology grade group. DHT = dihydrotestosterone.

Since it has been reported that natural colibactin and colibactin 742 both induce DNA crosslinking, we postulated that colibactin 742 would cause replication stress by stalling replication fork progression [9,19]. We used DNA fiber assays to test whether colibactin 742 would affect progression of replication forks and restarting of stalled replication forks. We found that colibactin 742 significantly increased the abundance of stalled forks (*p* < 0.1; Fig. 6A).

PC may result from aberrant translocation involving an ERG transcription factor. Fusion of unrepaired stalled replication forks is a major source of translocations. If repair of stalled replication forks fails, free DNA ends in repair intermediates, such as reversed and cleaved forks, can be aberrantly ligated to another free DNA end from a distinct fork to generate chromosome translocations or other rearrangements [61]. Adjacent forks on the same fiber with asymmetric sizes and asymmetric CIdU:IdU-labeled tracks represent a subset of RFF events. Using this method, we discovered that treated RWPE-1 prostate cells treated with colibactin 742 had 2.2-fold more asymmetrically sized and asymmetrically labeled forks on the same fiber (*p* < 0.01) than control cells (Fig. 6B). This implies that colibactin 742 treatment can cause RFF, which is a key source of chromosomal translocations.

## 4. Discussion

We hypothesized that synergy between colibactin 742 and DHT would lead to an increase in genomic instability. We observed a marked increase in somatic mutational density after combination treatment in vitro, which indicates higher levels of kataegis. Kataegis sites were distributed across the genome and found near genomic rearrangements and CNAs [62]. Kataegis is associated with higher Gleason score and defects in DNA damage repair [60]. Our results suggest that combination treatment in cell culture has the most significant effect on genome instability and promotes processes associated with higher tumor grade.

Natural colibactin derived from *pks*^+^ *E. coli* is highly unstable [63]. Therefore, we used the stable analog colibactin 742, which recapitulates the crosslinking activity of the bacterial metabolite [19]. Our findings suggest that colibactin 742 contributes to initial tumorigenesis in the prostate, consistent with colorectal cancer, for which the colibactin mutational process occurs early in carcinogenesis [4]. Given the structural similarity of colibactin 742 and natural colibactin, these findings are likely to apply to natural colibactin from *pks*^+^ *E. coli* as well, although this hypothesis remains to be tested.

Colibactin is a potential factor in PC etiology via exacerbation of the characteristic *TMPRSS2:ERG* fusion that is present in 50–60% of PCs of European ancestry. We did not find evidence of *TMPRSS2:ERG* fusion in cells treated with colibactin 742. However, combination treatment did induce many SVs. The pattern of SVs induced by combination treatment suggests chromoplexy as events are distributed across many chromosomes and number in the dozens [64]. It has been reported that chromoplexy is present in 50–90% of all prostate tumors.

Colibactin 742 causes replication stress by stalling replication forks, leading to an increase in unrepaired RFF events. These data are consistent with findings that other crosslinking agents induce replication fork stalling and chromosomal translocations [65]. While the pathway for repair of colibactin crosslinks is still unclear, it is likely that the Fanconi’s anemia (FA) crosslink repair pathway plays a role, as it is the major crosslink repair pathway protecting the human genome [9,66]. In addition, FA patients present with massive chromosomal fusion events observed in bone marrow, consistent with the fork data seen here.

Our study has several limitations. Our retrospective clinical analysis was strictly observational and precludes causality. We did not use *pks*^+^ *E. coli* to dose colibactin, and instead used the stable analog colibactin 742. In addition, in vitro analyses were conducted using two biological replicates in a cell line, which limits statistical power and may not fully recapitulate the complexity of PC etiology. It is possible that other factors further mediate the impact of colibactin and androgens on tumor initiation and/or progression. Future in vitro analyses may focus on additional PC model systems to fully characterize this interaction across a diversity of genetic and epigenetic landscapes, and in the context of more diverse behavioral and mutagenic life histories.

Our findings provide evidence supporting a potential role for colibactin-producing *pks*^+^ *E. coli* in PC etiology and synergy between colibactin and DHT in driving tumorigenesis via acceleration of genome instability and localized hypermutation.

## 5. Conclusions

In summary, we observed a significant association between the presence of the colibactin-producing *pks* gene island and PC diagnosis in a 235-patient clinical cohort. Results from in vitro trials using the synthetic colibactin 742 alone and in combination with DHT suggest a synergistic effect on accelerating genome instability and hypermutation.

Future investigations are warranted to assess the effects of colibactin across a broader spectrum of patient populations. The impact of colibactin among individuals with varying genetic risk remains unclear. Large-scale clinical studies with ancestrally diverse cohorts are crucial for understanding the impact of colibactin among individuals with varying genetic risk profiles. Clinical trials assessing whether colibactin exposure in patients with high genetic risk affects active surveillance decision-making could potentially refine existing risk stratification frameworks.

It remains unclear if patients with colibactin exposure are more sensitive to androgen-ablating therapies. Molecular correlation analyses in large clinical trials and additional studies of the downstream signaling consequences of androgen ablation in the presence and absence of *pks* gene islands would help in establishing the potential of this therapeutic strategy. Notably, androgen ablation may improve survival in *pks*^+^ patients by mitigating the hypermutation phenotype we have described. Our findings highlight the potential role of colibactin in PC tumorigenesis and underscore the need for further research to explore its potential as a biomarker and therapeutic target.

## Supplementary Material

Supp Data 2

Supp Data 1

Supp Data 3

Supp Data 4

Supp Data 5

Supplementary Information

Supp Data 6

Supp Data 7

## Figures and Tables

**Table 1 –:** Demographic data for the clinical study cohort

Parameter	Cancer absent (*n* = 79)	Cancer present (*n* = 156)	*p* value
Mean age, yr (standard deviation)	63.9 (9.04)	65.5 (8.38)	0.171
Median age, yr (range)	65.0 (44.0–81.0)	67.0 (37.0–81.0)	
Race, *n* (%)			
American Indian or Alaskan Native	1 (1.3)	3 (1.9)	0.267
Asian-American or Pacific Islander	0 (0)	2 (1.3)	
Black	35 (44.3)	52 (33.3)	
Other	10 (12.7)	12 (7.7)	
Unknown	1 (1.3)	5 (3.2)	
White	32 (40.5)	82 (52.6)	
Ethnicity, *n* (%)			
Hispanic	11 (13.9)	28 (17.9)	0.398
Non-Hispanic	63 (79.7)	123 (78.8)	
Unknown	5 (6.3)	5 (3.2)	
Diabetes, *n* (%)			
Absent	61 (77.2)	101 (64.7)	0.072
Present	18 (22.8)	54 (34.6)	
Data missing	0 (0)	1 (0.6)	
Mean body mass index, kg/m^2^ (standard deviation)	30.1 (5.21)	29.6 (5.12)	0.508
Median body mass index, kg/m^2^ (range)	29.0 (21.4–43.1)	29.0 (18.7–43.7)	
Data missing	0 (0)	4 (1.3)	
Digital rectal examination, *n* (%)			
Abnormal	7 (8.9)	51 (32.7)	<0.001
Normal	60 (75.9)	98 (62.8)	
Data missing	12 (15.2)	7 (4.5)	
Mean PSA, ng/ml (standard deviation)	8.07 (4.03)	12.2 (15.5)	<0.001
Median PSA, ng/ml (range)	6.76 (2.23–19.3)	7.21 (0.140–133)	
ISUP grade group, *n* (%)			
Grade group 1	0 (0)	58 (37.2)	
Grade group 2	0 (0)	38 (24.4)	
Grade group 3	0 (0)	21 (10.9)	
Grade group 4	0 (0)	17 (7.7)	
Grade group 5	0 (0)	12 (6.4)	

ISUP = International Society of Urological Pathology; PSA = prostate-specific antigen.
