# Supplementary material for: Colibactin Exerts Androgen-dependent and -independent Effects on Prostate Cancer
Source: Eur Urol Oncol. Author manuscript; Available in PMC 2026 Jun 1. (PMC12075626; doi:10.1016/j.euo.2024.10.015)

Supplementary Figure 1

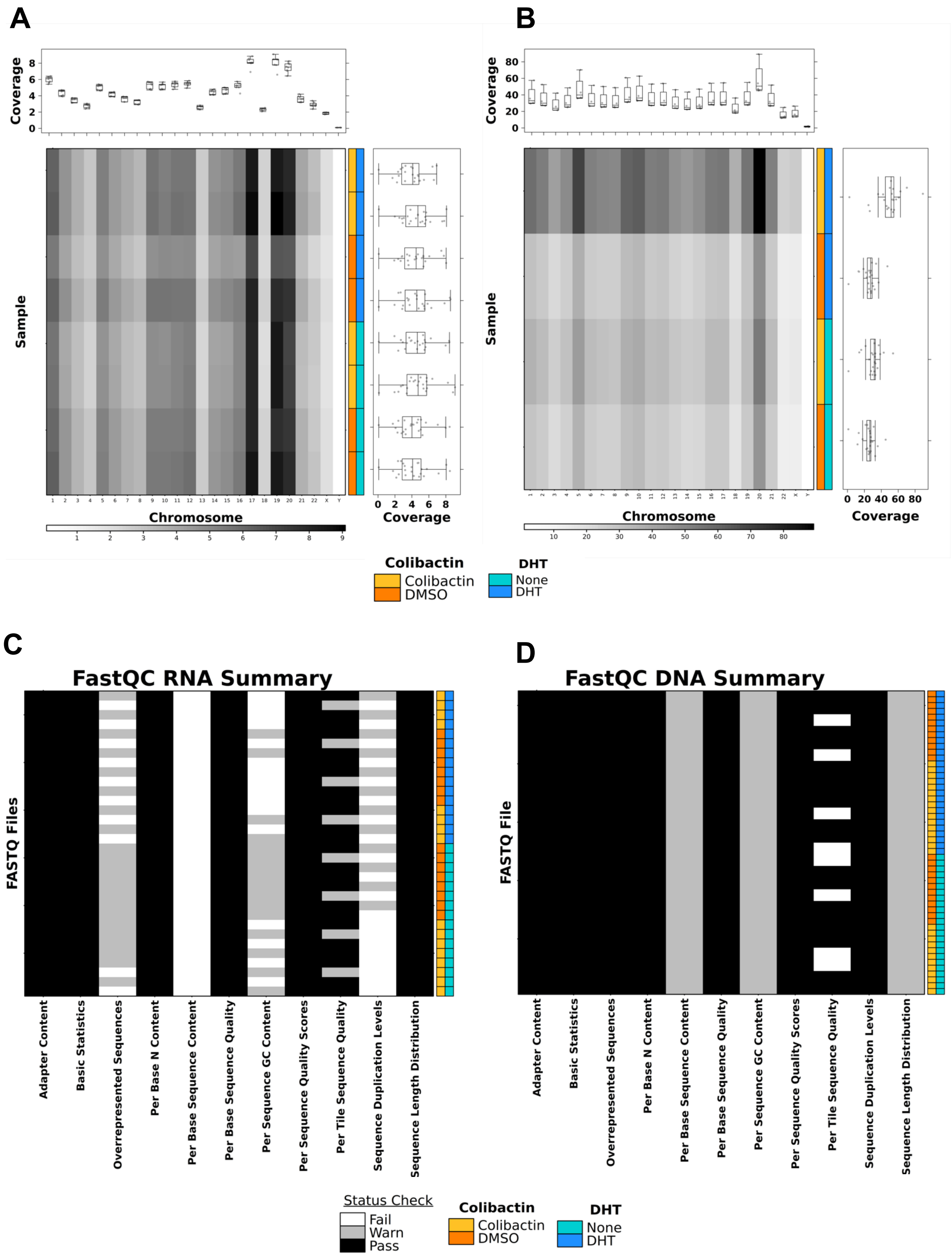

Supplementary Figure 2

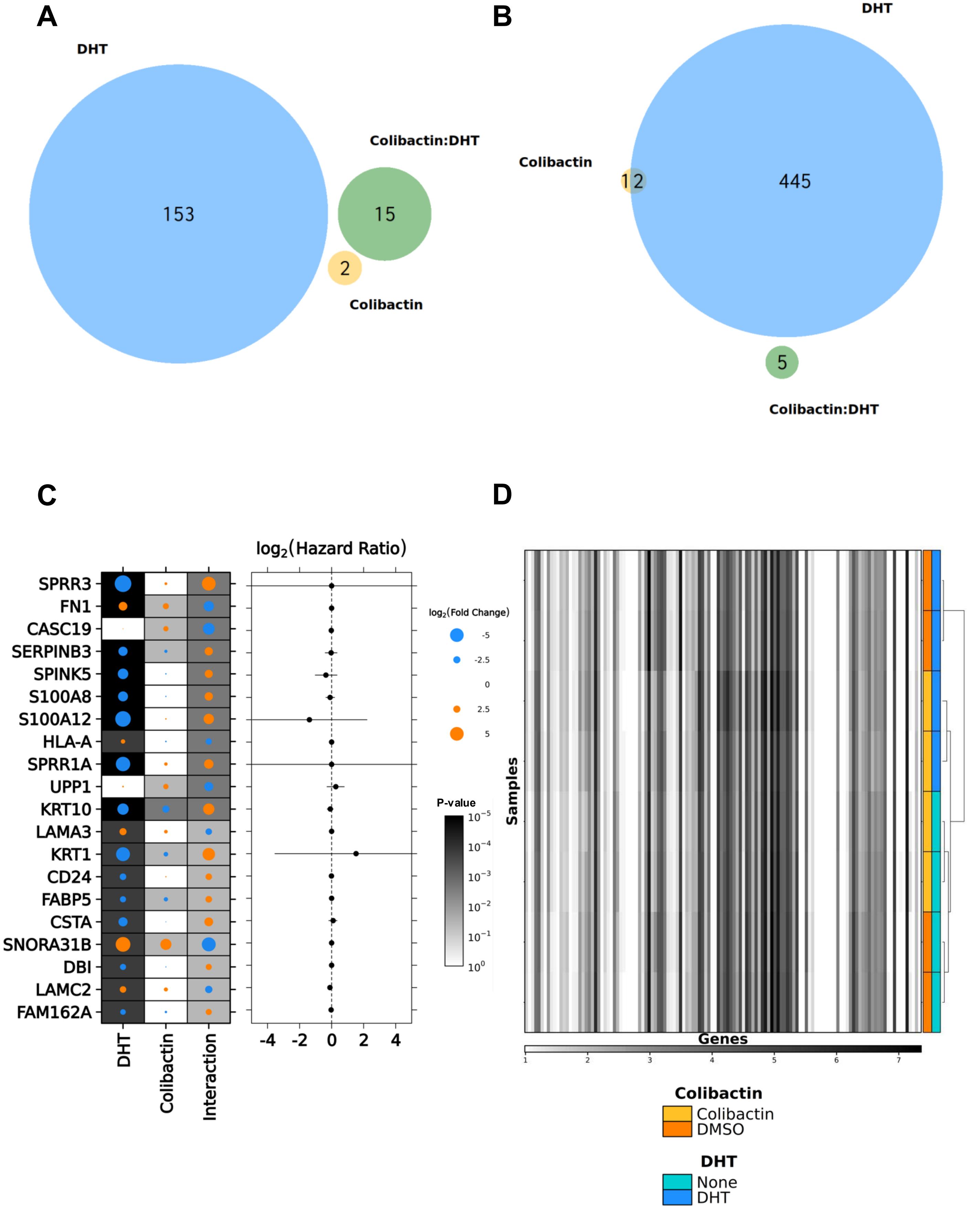

Supplementary Figure 3

A

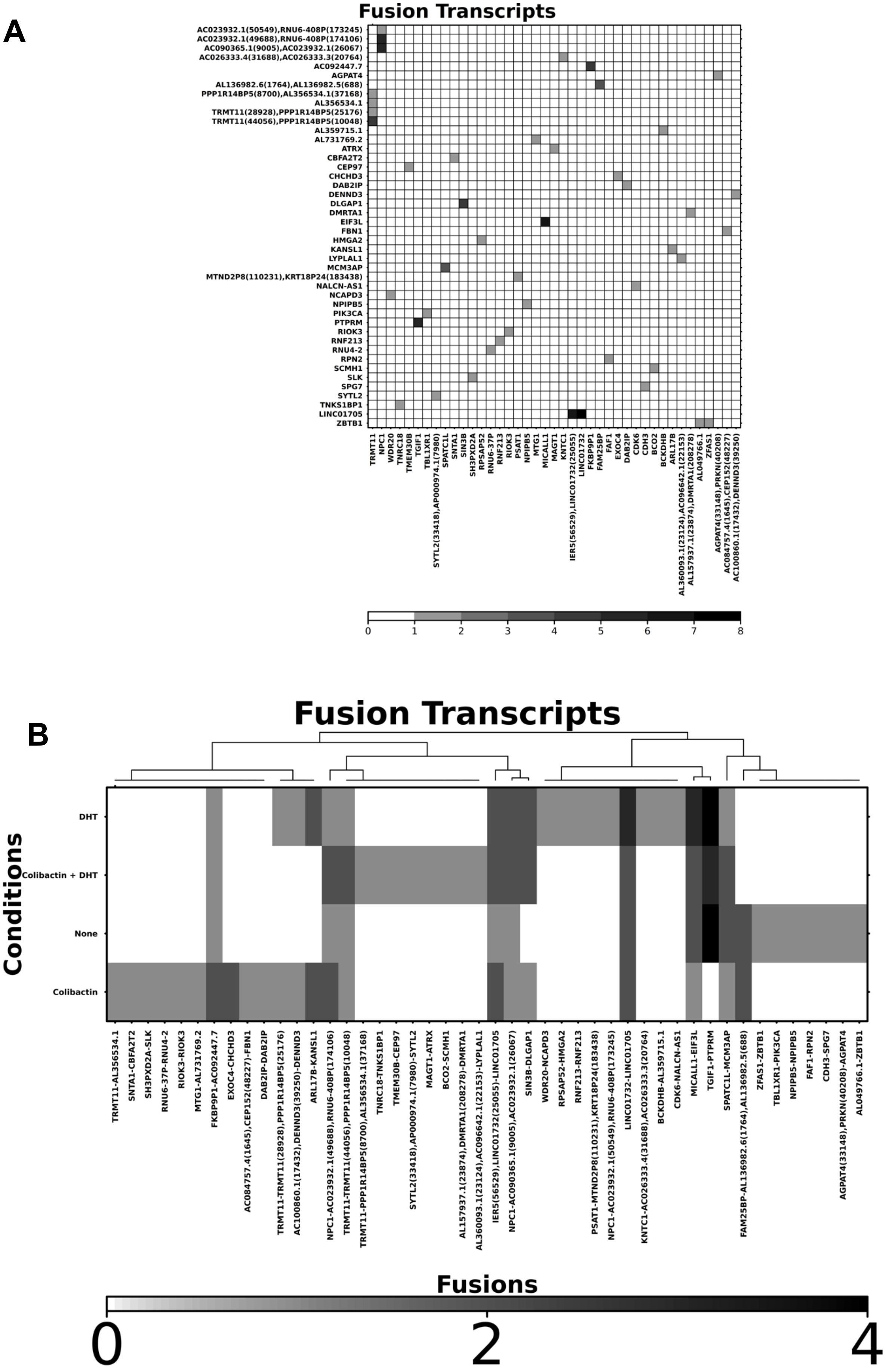

B

Supplementary Figure 4

A

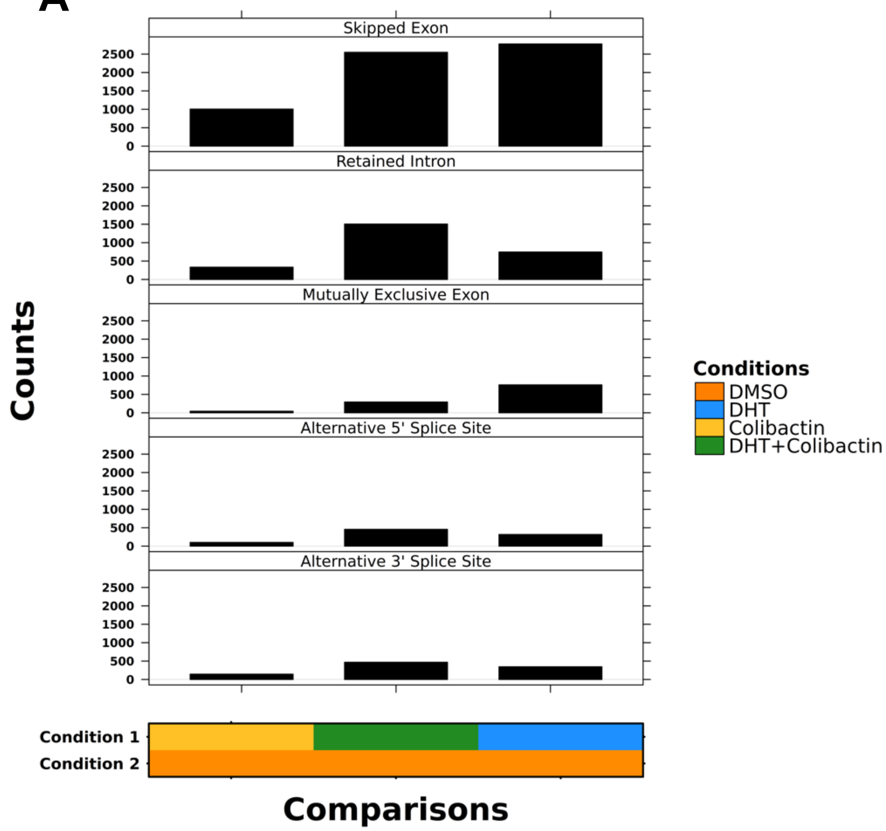

B

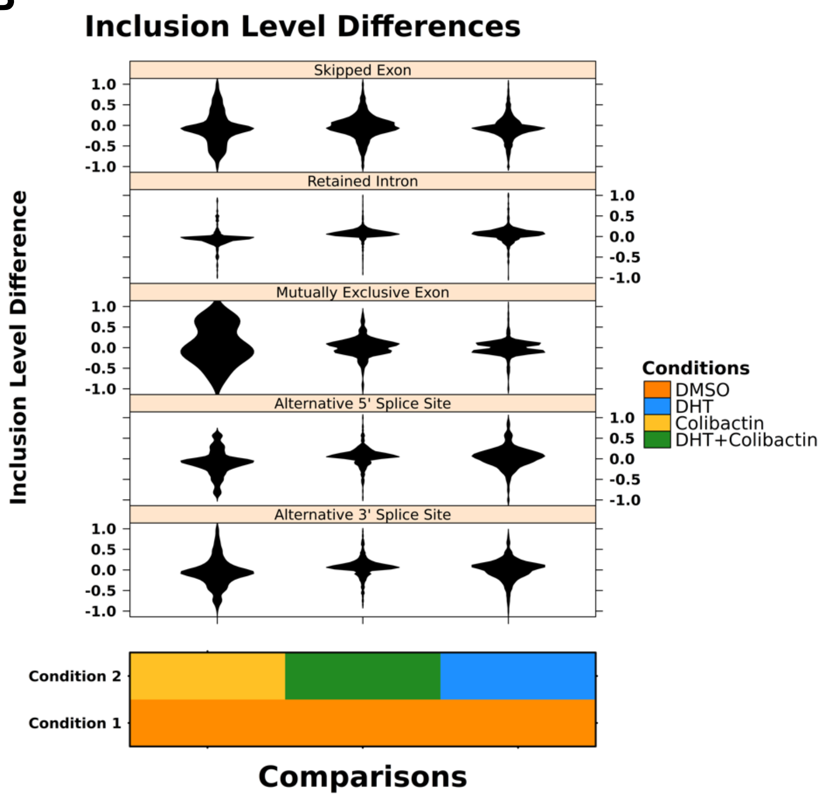

Supplementary Figure 5

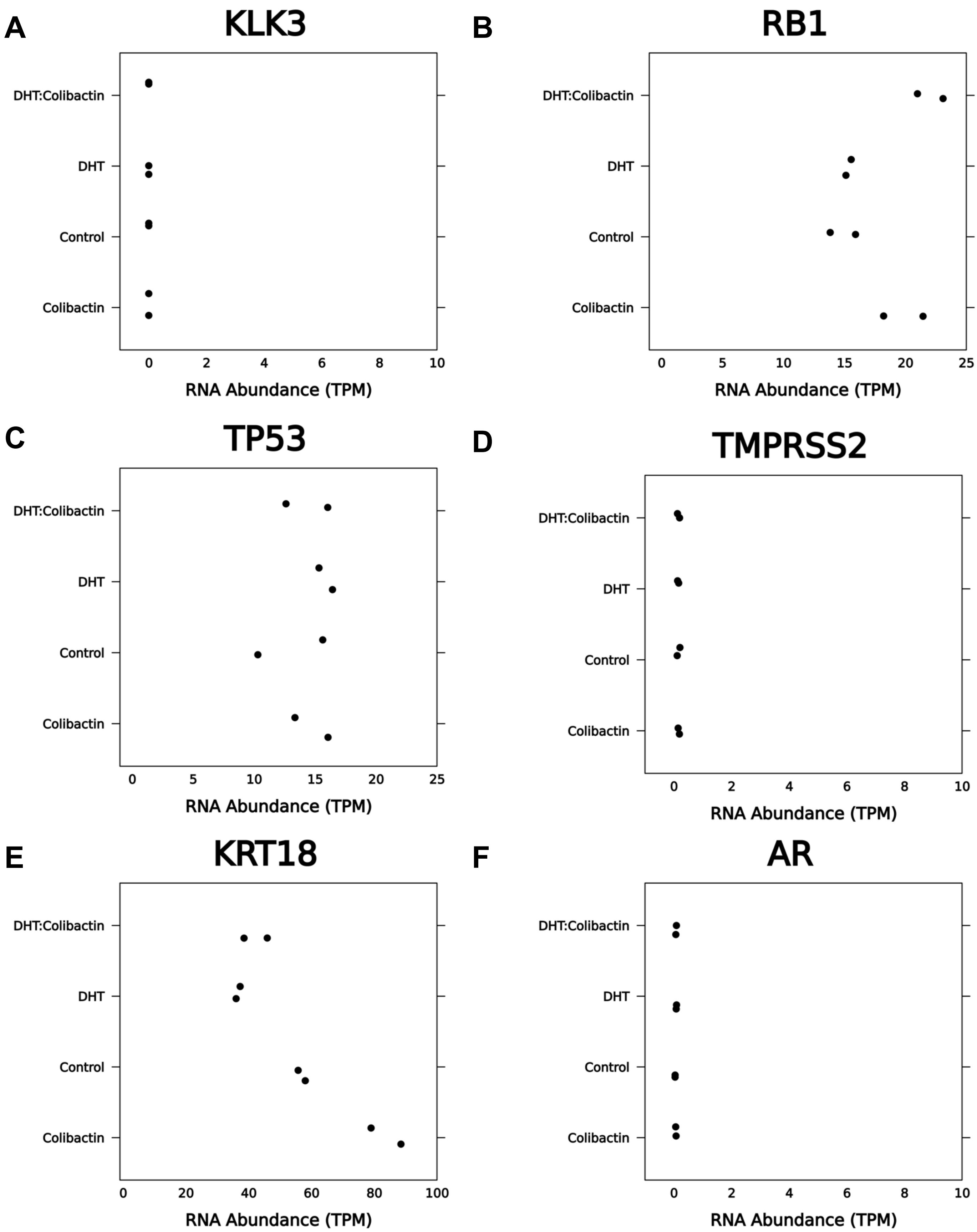

Supplementary Figure 6

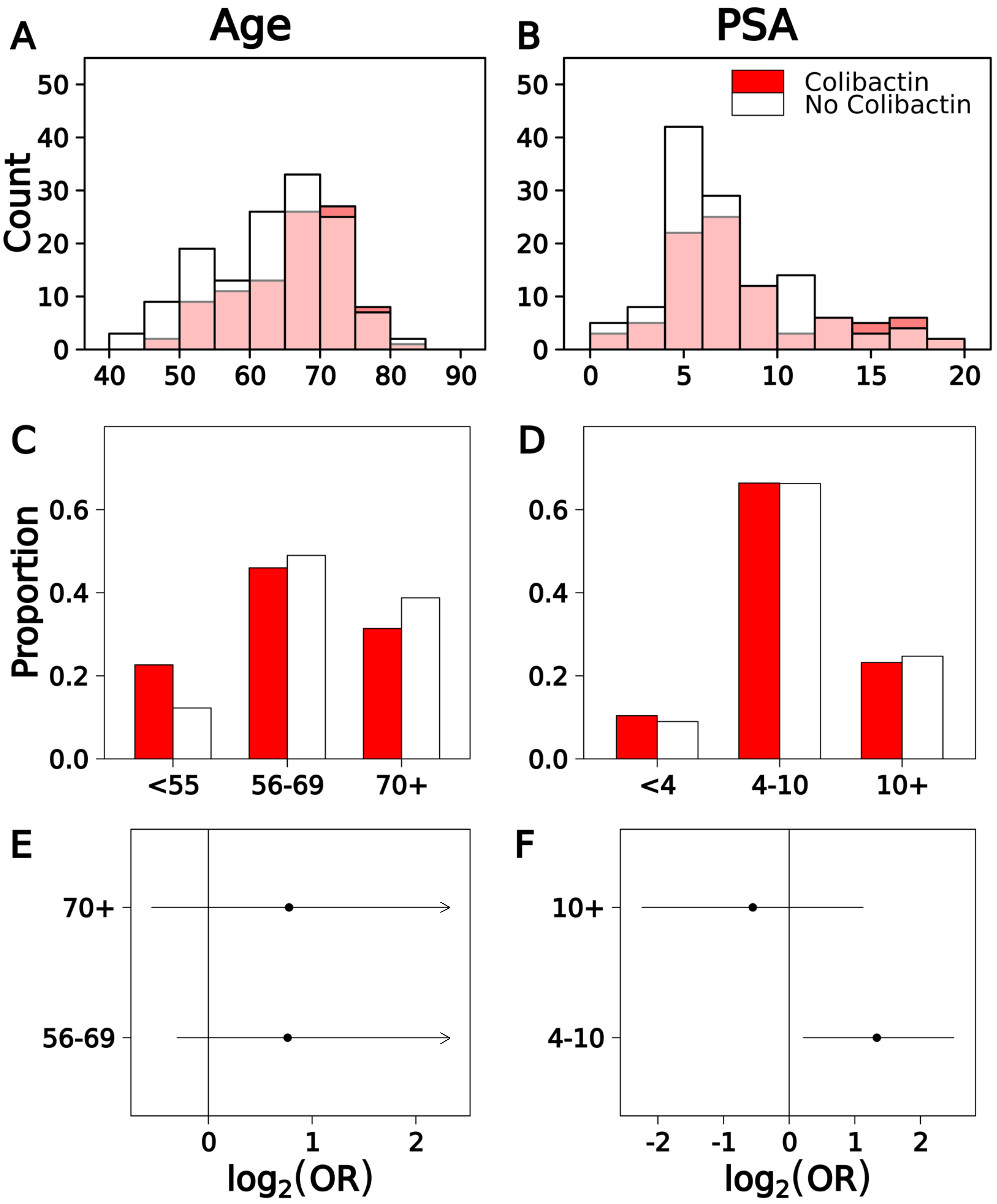

Supplement: Supplementary Information [file NIHMS2043305-supplement-Supplementary_Information.pdf]
